# SuperLigands – a database of ligand structures derived from the Protein Data Bank

**DOI:** 10.1186/1471-2105-6-122

**Published:** 2005-05-19

**Authors:** Elke Michalsky, Mathias Dunkel, Andrean Goede, Robert Preissner

**Affiliations:** 1 BCB (Berlin Center for Genome Based Bioinformatics) at Institute of Biochemistry, Charité (University Medicine Berlin), Monbijoustr. 2, 10117 Berlin, Germany

## Abstract

**Background:**

Currently, the PDB contains approximately 29,000 protein structures comprising over 70,000 experimentally determined three-dimensional structures of over 5,000 different low molecular weight compounds. Information about these PDB ligands can be very helpful in the field of molecular modelling and prediction, particularly for the prediction of protein binding sites and function.

**Description:**

Here we present an Internet accessible database delivering PDB ligands in the MDL Mol file format which, in contrast to the PDB format, includes information about bond types. Structural similarity of the compounds can be detected by calculation of Tanimoto coefficients and by three-dimensional superposition. Topological similarity of PDB ligands to known drugs can be assessed via Tanimoto coefficients.

**Conclusion:**

*SuperLigands *supplements the set of existing resources of information about small molecules bound to PDB structures. Allowing for three-dimensional comparison of the compounds as a novel feature, this database represents a valuable means of analysis and prediction in the field of biological and medical research.

## Background

Protein modelling and structure prediction as well as binding and interaction prediction have become very valuable instruments for researchers in biology and medicine. In order to build reasonable and useful models, as much information as possible has to be incorporated into the protein modelling process. To refine protein models, chemical as well as spatial information about ligand structures can be considered, specifically to optimise side-chain conformations around binding-sites [1].

Several databases delivering structures and different additional information about ligand molecules from the Protein Data Bank (PDB) [2], [21] have been provided on the Internet. Ligand Depot [3], [22] comprises chemical and structural information for small molecules found in the PDB and also provides a graphical interface for performing chemical substructure searches. Idealized three-dimensional structures and additional information about PDB ligands can be retrieved via the search interface of the E-MSD macromolecular structure relational database [4], [23]. Besides many other features, Relibase [5], [24] allows for two-dimensional similarity and substructure search among the ligands as well as for sequence similarity search among the corresponding proteins. LigBase [6], [25] is a database of ligand binding sites aligned with related protein structures and sequences. Various information about ligands bound to macromolecules deposited in the PDB can be retrieved from many further sources like HIC-Up [7], [26], PDBsum [8], [27] and the IMB Jena Image Library of Biological Macromolecules [9], [28]. The latter can be searched after geometrical properties of the ligand binding sites.

Information contained in these databases can help identifying ligands which are likely to bind to a given protein structure. The opposite question, namely to find target proteins for a certain ligand, was addressed in [10], where a collection of protein active sites was extracted from the PDB and scanned with aid of a docking algorithm. Further data collections emphasize the link between binding affinities and structures of the protein-ligand complexes and, inter alia, provide experimentally measured binding data, e.g. PLD [11], [29] LPDB [12], [30], PDBbind [13], [31].

For modelling and simulation purposes, chemical and spatial information about protein ligands is vitally important. Addressing this fact, *SuperLigands *is a collection of small molecule structures contained in the PDB, facilitating comparison of the molecules regarding their two-dimensional similarity. As spatial comparison of compounds can deliver valuable information in addition to this, *SuperLigands *also allows for three-dimensional superpositions. Spatial coordinates of the compounds can be retrieved as MDL Mol files, which include information about multiple bonds.

## Construction and content

Native conformations of small molecules contained in the PDB and additional information were collected from the PDB [2], [21], Ligand Depot [3], [22] and MSD [4], [23] and deposited in the database *SuperLigands*. The database has been designed as a MySQL relational database and supplemented with a user-friendly web interface. Database queries are performed and HTML pages are generated via PHP scripts. The freely available MDL^® ^Chime plug-in is used to display molecules and allows the user some manipulations of the view and to store the displayed molecule in the MDL Mol file format.

In order to enable fast two-dimensional searches, 960 bit binary fingerprints (MDL MACCS Keys [14]) were calculated and stored in the database for all ligands. Tanimoto coefficients are calculated via a PHP script. Here, all 960 MDL keys are included and equally weighted. The Tanimoto coefficient for two structures a and b is defined as follows: T(a,b)=Nab/(Na+Nb-Nab), where Na and Nb are the numbers of bits set in the fingerprint of structure a and b, respectively, and Nab is the number of bits which are common to both fingerprints.

Three-dimensional superposition of two different PDB ligands is performed in the following way: each conformation of one molecule occurring in the PDB is superposed with each conformation of the second molecule. Those two instances matching best are displayed. The best match is defined by maximizing the *score *defined by



where RMSD is the Root Mean Square Deviation of the superposed atoms. For completion, PDB codes, chain identifiers and positions in the PDB files of the matched conformations as well as the atom numbers of both ligands, the number of superposed atoms, the number of superposed atoms of the same type and the RMSD of the superposition are returned. For detailed information regarding the superposition algorithm see [15].

## Utility

*SuperLigands *can be searched by hetero-ID (i.e. the three-letter PDB code for hetero-compounds), name, molecular formula or PDB identifier. In the results table, hetero-ID and names of the compounds are given. Moreover, the molecular structure is displayed in one cell of the table where it can be rotated by the user and different displaying options can be chosen. More information like molecular formula, atom numbers and occurrence in the PDB can be retrieved additionally.

The database SuperLigands contains compounds defined by 'HETATM' records in PDB files. Some of these molecules may be bound to pseudopeptides but can also be separate ligands. Coming across such a molecule, the user is given a hint and is provided with a list of pseudopeptides in which this molecule is bound.

The user can search the database for molecules that have a significant two-dimensional similarity to a given ligand or assess the three-dimensional similarity of two compounds by superposing them with each other. Such similarity queries can be performed starting from the search results tables or directly using separate forms. Using such a form, the Tanimoto coefficient of two given structures can also be retrieved.

A typical example for a query to *SuperLigands *is a search for tobramycin, known as antiinfective and antibacterial drug, starting in the main form. Searching the database for similar compounds in the next step supplies the drug kanamycin as PDB ligand with the highest Tanimoto similarity (98.6%). Now, a three-dimensional superposition of all instances of tobramycin (32 atoms, two instances) and kanamycin (33 atoms, four instances) occurring in the PDB can be performed. The best fitting structures are superposed and then displayed. In this case, best fitting are the instances of tobramycin in PDB entry 1m4d and kanamycin in 1m4i with an RMSD of 0.14Å (32 atoms superposed). Navigating through the website, the topological and spatial similarities of PDB ligands can be obtained easily. For example, tobramycin and ribostamycin (31 atoms, four instances) have a Tanimoto coefficient of 96.5%, the RMSD of their best superposition is only 0.95Å (25 atoms superposed). In turn, geneticin (34 atoms, five instances) delivers a Tanimoto coefficient of only 81.1% and a much better RMSD (0.16Å, 30 atoms superposed).

**Figure 1:**
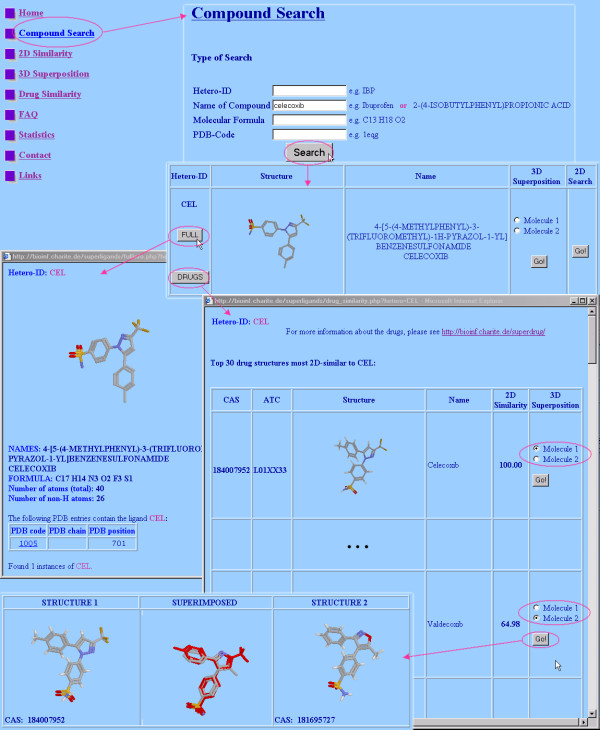
**Usage of the web interface of *SuperLigands***. From the main menu, the form *Compound search *can be reached. Here, a PDB ligand can be searched after hetero-ID, name, molecular formula or PDB identifier. In the first column of the results table, two buttons can be found to retrieve more information. The *FULL info *button delivers detailed information about the selected PDB ligand like molecular formula, atom numbers and occurrence in the PDB. After clicking the *DRUGS *button, a two-dimensional similarity search among the drugs in the *SuperDrug *database [16] is performed. The best hits are displayed in a new window. From here, they can be spatially superposed. In the figure, this procedure was carried out for celecoxib, a COX-2 inhibitor which was recently categorised as problematic (see the "Pfizer Statement on New Information Regarding Cardiovascular Safety of Celebrex" [18]). The two-dimensional similarity search in the *SuperDrug *database delivers only hits below 72% Tanimoto similarity. A following spatial superposition of the best hits reveals a further COX-2 inhibitor, namely valdecoxib, (RMSD 0.26Å and 21 of 22 atoms superposed) as very similar to celecoxib. The Tanimoto similarity of celecoxib and valdecoxib is only 65% and there are two drugs more similar to celecoxib: Sulfaphenazole (71% Tanimoto similarity, spatial superposition with 0.65Å RMSD and 15 of 22 atoms superposed) and Sulfamazone (67% Tanimoto similarity, spatial superposition with 0.32Å RMSD and 17 of 26 atoms superposed). Nevertheless, the three-dimensional comparison here proves to be very important to reveal molecular similarities in addition to topological comparison (as also shown in the example in the section *Utility*), which is confirmed by the fact that valdecoxib was categorised as toxic [19] and sales of this drug were suspended recently [20].

As an additional feature of *SuperLigands*, similarity of PDB ligands to known drugs can be assessed in a comfortable manner. Starting with a ligand, a two-dimensional similarity search as described above can be initiated, not only among the PDB ligands but also in a database containing the structures of known drugs (*SuperDrug Database *[16], [32]). The drug structures found can be superposed spatially (for an example, see Figure 1).

## Discussion

### Statistics: comparison of PDB ligands with drugs

Recently, a database containing 2396 drug molecules and having the same design as *SuperLigands *has been created (*SuperDrug Database *[16], [32]). To answer the question, how many drugs or drug-like molecules are bound to PDB structures, Tanimoto coefficients have been calculated for all pairwise combinations of molecules from *SuperLigands *and the *SuperDrug Database*. A set of 5,040 PDB ligands has been incorporated into these calculations. Considering two molecules having a Tanimoto coefficient of 100% (or greater than 95% ; 90%) identical or very similar, this analysis reveals that 413 (771 ; 1,457) of 5,040 PDB ligands are drugs or drug-like compounds.

**Figure 2:**
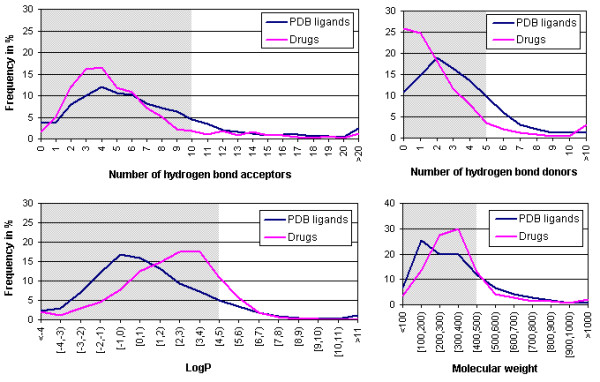
**Statistics: comparison of PDB ligands with drugs**. Chemical properties of 5040 PDB ligands and 2396 drug molecules are compared: histograms for numbers of hydrogen bond donors and acceptors, logP value and molecular weight are shown. Molecular weight within [100,200) means that the molecular weight is greater than or equal to 100 and less than 200. Those areas for which the Lipinski "Rule of 5" is fulfilled, are highlighted in grey.

Furthermore, some chemical properties of PDB ligands and drugs have been compared (see Figure 2). The distributions of numbers of hydrogen bond donors for PDB ligands and drugs differ most significantly. A bigger percentage of the drugs (26%) have no hydrogen bond donor, the largest fraction of the PDB ligands (19%) have two of them. About half of the drugs have no or only one hydrogen bond donor, which applies for only a quarter of the PDB ligands. About one third of the drugs have three or four hydrogen bond acceptors, the fractions of drugs with nine or more hydrogen bond acceptors drop below 3%. For the PDB ligands, the distribution is more flat: only 22% of them have three or four hydrogen bond acceptors, still over 3% of them have 11 hydrogen bond acceptors. Most drugs have a logP value around 3, and the logP values of the PDB ligands accumulate around the negative value -1. Approximately the same fraction of PDB ligands and drugs are "drug-like" according to the Lipinski "Rule of five" [17]: 92 and 91%, respectively, have a logP value less than 5, although altogether the logP values of the drugs are closer to this critical value. A majority of the PDB ligands have very low molecular weights in comparison to the drugs, which supposedly is be caused by the fact that in proteins often very small solvent molecules are bound. Nevertheless, slightly more (5%) drugs than PDB ligands fulfil the Lipinski "Rule of five" regarding the molecular weight. The same applies for the numbers of hydrogen bond donors (and acceptors): 7% (5%) more drugs fulfil the Lipinski "Rule of five".

Compounds violating more than one of the Lipinski Rules are assumed to have problems with bioavailability and are therefore presumably not suitable as drugs. Table 1 shows the percentages of PDB ligands and drugs violating the Lipinski Rules. From this table can be seen that a total of approximately 19% of the PDB ligands and 10% of the drugs, respectively, violate more than one of the Lipinski Rules. This analysis reveals that there are only marginal differences between PDB ligands and drugs regarding single chemical properties. But, not surprisingly, from a general point of view, PDB ligands are significantly less drug-like than drugs.

### Discussion

*SuperLigands *is a collection of PDB ligands freely accessible via a user-friendly web site. Molecular coordinates can be retrieved as MDL Mol files, supplementing the connectivity records contained in PDB files with bond types, which are necessary for modelling and simulation purposes. The database can be searched for compounds similar to a given ligand by comparison of Tanimoto coefficients. As stated in [15] and shown in the example in the section *Utility*, spatial comparison of small molecules can reveal more similarities, and thus similar kinds of interaction, than a pure two-dimensional topology comparison. With aid of *SuperLigands*, such three-dimensional comparisons can be performed easily. Moreover, the topological similarity of PDB ligand structures to known drugs can be assessed by calculation of Tanimoto coefficients.

## Conclusion

The database presented here supplements the set of existing resources of information about small molecules bound to PDB structures. As novel features, three-dimensional comparison of molecules as well as topology comparison of PDB ligands with known drugs are made possible. Thus, *SuperLigands *represents a valuable means of analysis and prediction in the field of biological and medical research.

## Availability and requirements

The database is publicly accessible at . For visualisation, the free browser plug-in MDL^® ^Chime is required. Chime runs on Windows systems with Microsoft Internet Explorer (6.0 or 5.5 SP2) or Netscape 4.75, 4.79 or on Mac OS 9.0 or 8.6 with Netscape 4.75 (please see  for detailed information).

## Authors' contributions

EM designed the database and the web site and finished its functionality, was responsible for data acquisition and processing and drafted the manuscript. MD delivered the basic part of the website functionality and contributed to database conception and data processing. AG provided the tool for three-dimensional superposition and helped to draft the manuscript. RP conceived of the project, and participated in its design and coordination and helped to draft the manuscript. All authors read and approved the final manuscript.

## Figures and Tables

**Table 1:** Percentage of PDB ligands and drugs violating certain numbers of Lipinski Rules

Number of violated Lipinski Rules	PDB ligands	Drugs
0	64.4	75.7
1	16.9	14.0
2	10.3	5.7
3	8.3	4.7
4	0.1	0.0
